# Serum PD-1 regulation and PD-1 expression of CD4+Foxp3+ regulatory T cells in patients in thyroid eye disease associated with immunosuppression treatment

**DOI:** 10.3389/fopht.2024.1491053

**Published:** 2024-12-16

**Authors:** Atsushi Sakai, Mizuki Tagami, Norihiko Misawa, Yusuke Haruna, Mami Tomita, Shigeru Honda

**Affiliations:** Department of Ophthalmology and Visual Sciences, Graduate School of Medicine, Osaka Metropolitan University, Osaka, Japan

**Keywords:** thyroid eye disease, immune checkpoint molecules, programmed cell death 1, flow cytometry analysis, regulatory T cell

## Abstract

**Purpose:**

Thyroid eye disease (TED) primarily occurs in hyperthyroid patients, sometimes resulting in poor visual prognosis. Although other autoimmune diseases have been reported to be associated with serum programmed cell death 1 (PD-1), the relationship with TED remains unknown. This study investigated the relationship between TED and immune checkpoint molecules.

**Methods:**

Serum immune checkpoint molecules were measured in TED and control patient blood samples. In TED patients, blood samples were compared before and 6 months after steroid pulse treatment. Cytometry analysis was additionally performed in TED and control patients to compare the expression of (PD-1) of T cells.

**Results:**

Serum concentrations of PD-1 in TED and control patients were 163.49 ± 79.01 (pg/mL) and 123.58 ± 46.61 (pg/mL) (*P* = 0.03). Serum PD-L1 concentration in TED was 157.89 ± 55.34 (pg/mL), while 152.58 ± 22.70 (pg/mL) in control patients (*P* = 0.92). For flow cytometry analysis, the mean fluorescence intensity (MFI) ratio of PD-1 in Foxp3high CD45RA- of the CD4+ T cells and CD127-CD25high of the CD4+ T cells were higher in TED versus control patients (*P* = 0.04, *P* = 0.02). There was also a higher percentage of PD-1 expressions on CD4+ T cells and Foxp3high CD45- T cells in TED patients versus that for control patients (*P* < 0.001, *P* = 0.003).

**Conclusions:**

PD-1 expression of CD4+Foxp3+ regulatory T cells appear to be associated with TED pathogenesis before and after treatment. Regulatory T cells expressed PD-1 have possibilities of clinical activity and autoimmune pathology of TED.

## 1 Introduction

Thyroid eye disease (TED) is an eye disease that is often presented in conjunction with hyperthyroidism, such as Basedow disease and Graves’ disease. While this is usually seen in patents with Graves’ disease (80%), it is also found in patients with autoimmune hypothyroidism due to Hashimoto’s thyroiditis (10%), and in patients without thyroid disease (10%) (1). TED causes eye problems in 25-50% of people with thyroid dysfunction, and 3-5% of them develop severe disease (2). A previous meta-analysis and systematic review that evaluated the prevalence of TED in Graves’ disease, reported it was 38% in Europe, 44% in Asia, 27% in North America and 58% in Oceania (3).

TED can sometimes become a sight-threatening illness. For example, cases with compressive optic neuropathy and corneal ulcers have been classified as being severe TED. Furthermore, several treatments have been reported for cases with compressive optic neuropathy and corneal ulcers that have been classified as severe TED, such as intravenous methylprednisolone and/or orbital radiotherapy, teprotumumab, and rituximab (4).

In TED patients, interactions between major histocompatibility complex class II (MHC II), self-antigens, and T cell receptors have been shown to increase intercellular adhesion molecule-1 (ICAM-1), nuclear factor (NF)-κβ nuclear translocation, interleukin (IL)-1, IL-6, and IL-8, resulting in orbital T cells promoting fibroblast proliferation (5, 6). Orbital fibroblasts upregulate the proinflammatory cytokines IL-1, and transforming growth factor (TGF)-β, which increases hyaluronic acid synthesis, that then sustains orbital inflammation (7). TED-derived IgG and insulin-like growth factor-1 (IGF-1) upregulate normal T-cell expression and increase T-cell migration to the orbit. These changes result in inflammation and fibrosis of the orbital tissue. In fact, IGF-1 receptor on fibroblasts in TED is significantly increased compared to control patients (7).

In TED patients, inflammation of the extraocular muscles, adipose tissue and lacrimal gland occurs, resulting in exophthalmos, upper eyelid retraction and decreased tear secretion (8). Furthermore, these disorders lead to damage of eyelids, conjunctiva, cornea, eye movement and optic nerve, resulting in various symptoms. In severe cases, diplopia and reduced vision occur, significantly impairing quality of life (QOL). These symptoms occur commonly in hyperthyroidism patients, but they can also occur in people with euthyroidism or hypothyroidism (6).

It is known that the immune checkpoint molecules are inhibitory receptors that are expressed on the surface of the immune cells in order to prevent excessive immune reactions. A typical example is programmed cell death 1 (PD-1) which is detected by Ishida and his colleague in 1992 (9). There is a report that PD-1-deficient mice develop an autoimmune phenotype, and now PD-1 is known as an inhibitory receptor which controls immune responses (10).

Furthermore, immune checkpoint molecules (PD-1/PD-L1) have been implicated in various autoimmune diseases, like rheumatoid arthritis (RA) and systemic lupus erythematosus (SLE) (11). There are some reports that TED-like disease caused by immune checkpoint molecule inhibitors (12, 13). Therefore, it is possible that immune checkpoint molecules, especially PD-1/PD-L1, are involved in TED, but the relationship between TED and immune checkpoint molecules remains unclear.

So, in this study we measured and compared the immune checkpoint molecules between TED and healthy subjects. In addition, we also measured and compared the immune checkpoint molecules before and after steroid pulse treatment in TED patients.

## 2 Materials and methods

### 2.1 TED patient diagnosis

For the TED diagnosis, orbital magnetic resonance imaging (MRI) short TI inversion recovery (STIR) images were obtained, and blood sampling was used to measure thyroid stimulating antibody (TSAb). It has been previously reported that extraocular muscles exhibit hyperintensity in STIR images of the orbital MRI, and that TSAb levels are elevated in TED patients (14, 15). Based on these previous findings, subjects were diagnosed with TED if the MRI showed hypertrophy/hyperintensity of the extraocular muscles, orbital fat, and levator palpebral superior muscle, and if the blood sample showed elevated TSAb levels. Steroid pulse therapy was administered in severe cases with corneal ulcer or compressive optic neuropathy, and moderate TED with diplopia.

### 2.2 Inclusion and exclusion criteria

Study inclusion criteria for TED were as follows: [1] moderate or severe thyroid eye disease with symptoms such as diplopia or compressive optic neuropathy, with steroid pulse therapy required, and [2] no history of malignant neoplasm. Study inclusion criteria for the control patients were as follows: [1] no history of thyroid disease including TED, malignant neoplasm, and autoimmune disease, [2] patients who were hospitalized to treat other illnesses, such as cataract.

In this study, we excluded patients who were over 80 years old, as it has been reported that serum PD-1 in the blood tends to increase in people over the age of 80 years (16). We also excluded cases in which thyroid-stimulating antibodies (TSAb), which are generally elevated in TED, were below the normal value of 110%.

### 2.3 Sample collection

This study was a retrospective, observational, case series, and this study was conducted utilizing both TED and control patients. We collected blood samples from patients with TED and other patients, such as cataracts, who were admitted to our department from January 2021 to May 2023. Blood samples from these patients were used to measure the serum immune checkpoint molecules such as programmed cell death 1 (PD-1) and programmed cell death ligand 1 (PD-L1). We additionally collected blood samples from TED patients after they underwent steroid pulse therapy in order to measure the serum immune checkpoint molecules. All of the TED patients of this study underwent steroid pulse therapy at Osaka Metropolitan University Hospital, Japan. At our facility, since oral steroid therapy is normally continued for half a year after the original steroid pulse therapy, we collected blood samples again after the patients underwent half a year of steroid pulse therapy.

Flow cytometric analysis was also performed in TED and control patients who were hospitalized from January 2023 to May 2023.

We obtained Institutional Review Board (IRB)/Ethics Committee approval (Osaka Metropolitan University, Protocol Identification Number: 4339), and the described research adhered to the tenets of the Declaration of Helsinki. Written, informed consent was obtained from all patients before enrollment from January 2021 to May 2023.

### 2.4 Measurement of serum and flow cytometry analysis of immune checkpoint molecules

For measurement of serum immune checkpoint molecules, 10 mL blood was collected in a collection tube containing 0.5 mL of sodium citrate at a concentration of 0.105 moles. Subsequently, the blood collection tube was then centrifuged at 5000 rpm for 5 minutes, after which the serum sample was collected. The collected serum was cryopreserved in a freezer at -80°C. These samples were sent to Sysmex Corporation (Wakinohamakaigandori Chuo Ward, Kobe, Hyogo Prefecture) for analysis, with serum PD-1 and PD-L1 measured using an HISCL-800 (Sysmex Corp.).

For the blood flow cytometry analysis, all samples were sent to the Clinical Flow Cytometry Laboratory of Tokyo University. All samples that underwent flow cytometry test were evaluated using a FACS Aria SORP (Japan Becton Dickinson Company, Minato Ward, Tokyo, Japan), using the FlowJo software (Japan Becton Dickinson).

The procedure involved separating PBMC from whole blood, washing twice, and determining the number of cells using by hemocytometer (approximately 1-10x10^6 cells/tube). 1ul of FVS700 was added to the sample, mixed well, and incubated on ice for 15 minutes. After that, an antibody for the surface antigen was added, washed, and incubated on ice for 15 minutes again. Next, as a permeabilization process, after removing the supernatant, 1ml of Fix/Perm working solution was added and incubated for 45 minutes at room temperature in the dark. Then, the sample was washed with 2ml of x1 Permiabilization buffer, and 100ul of x1 Permiabilization buffer and 2ul of Rat serum were added. The sample was incubated for 15 minutes at room temperature in the dark again, and an antibody for the intracellular antigen was added as appropriate. For intracellular Foxp3 staining, Thermo Fisher’s invitrogen #72-5776-40 eBio Anti-Human FoxP3 staining Set PE (catalog number: 72-5776-40) was used.

**Figure 1:**
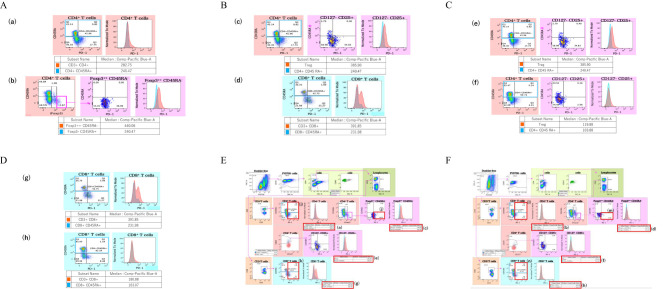
Sample results for the blood flow cytometry analysis in control and TED patients. Results of PD-1 MFI ratio on T cells. **(A)** It shows MFI ratio of CD4+ T cells, and (a) shows MFI of a TED patient, (b) shows MFI of a control patient. **(B)** It shows MFI ratio of CD4+ Foxp3 high T cells, and (c) shows MFI of a TED patient, (d) shows MFI of a control patient. **(C)** It shows MFI ratio of CD4+ CD127- CD25+ T cells, (e) shows MFI of a TED patient, (f) shows MFI of a control patient. **(D)** It shows MFI ratio of CD8+ T cells, and (g) shows MFI of a TED patient, (h) shows MFI of a control patient. Results of PD-1 expression on T cells in TED patient. **(E)** (i) shows PD-1 expression of the CD4+ T cells, (j) indicates PD-1 expression of the Foxp3high CD45- T cells, and (k) shows PD-1 expression of the CD8+ T cells in TED patient. Results of PD-1 expression on T cells in control patient. **(F)** (l) shows PD-1 expression of the CD4+ T cells, (m) indicates PD-1 expression of the Foxp3high CD45- T cells, and (n) shows PD-1 expression of the CD8+ T cells in control patient.

Regarding the flow cytometry results, viable cells were extracted by gating using FVS700, first. Lymphocytes and monocytes were separated from the SSC-A vs CD4 results, and then T cells were extracted using CD3 as a marker (see **Figures 1E, F**). After that, CD4-positive and CD8-positive T cells were extracted, then MFI ratio and PD-1 expression level in each cell were investigated. About the MFI ratio, we determined and calculated it by dividing the fluorescence intensity of effector T cells by the fluorescence intensity of naive T cells on relevant T cells. Foxp3 is generally used as a marker for regulatory T cells (Treg), but as previously reported, CD4+ CD25+ CD127low/- T cells express the highest amount of Foxp3, so we considered this cell as Treg in this study (17).

### 2.5 Statistical analysis

All results are reported as the means ± standard deviation. All collected data were entered into a Microsoft Excel spreadsheet (Microsoft, Redmond, WA) for further compilation and analysis. Statistical analyses were performed using EZR (Saitama Medical Center, Jichi Medical University; Kanda, 2012). We used the Mann-Whitney U test to compare the serum immune checkpoint molecules between the TED and control patients, and compare it before and after steroid pulse treatment. Regarding the gender ratio between each group, Fisher’s exact test was used to examine whether there was a significant difference.

A *P*-value < 0.05 was considered statistically significant.

## 3 Results

### 3.1 Evaluation and comparison of TED and control patients for PD-1 and PD-L1

**Figure 2:**
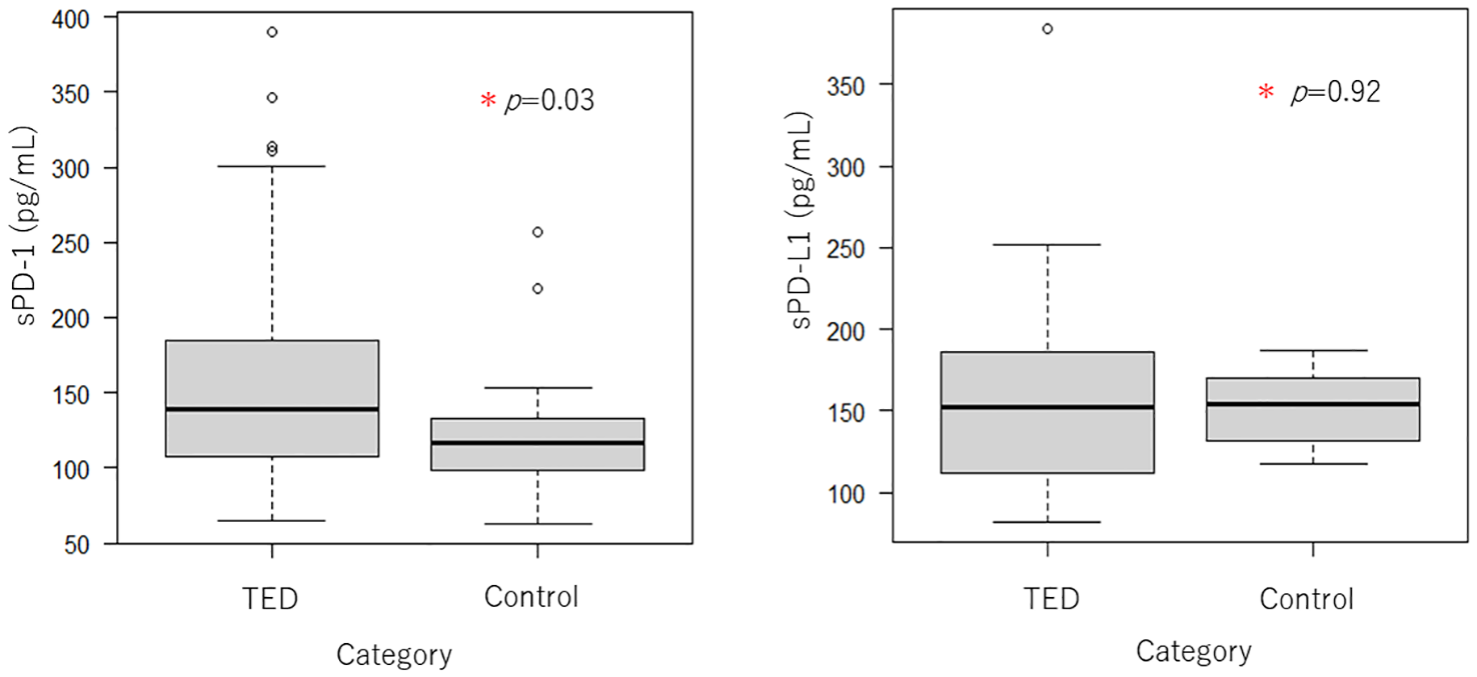
Boxplots of serum PD-1 and PD-L1 concentrations between TED (n = 44) and control patients (n = 19). Serum PD-1 was significantly higher in TED patients as compared to control patients, although there no significant difference in PD-L1.

As shown in **Table 1**, 44 total TED patients and 19 control patients were included in this study. There were 12 males and 32 females in the TED group, and 9 males and 10 females in the control patient group, there was no significant difference in gender ratio between each group (*P* = 0.15). 36 patients had hyperthyroidism, 1 patient had hypothyroidism, and 7 patients were euthyroidism as the underlying diseases in the TED group. The mean age of all TED patients was 56.06 ± 14.35 (range 29-79) years, while it was 50.68 ± 18.29 (range 27-79) years in control patients, and there were no significant differences between the groups. **Figure 2** shows boxplots of serum PD-1 and PD-L1 concentrations between TED and control patients. PD-1 concentration in the TED patients was 163.49 ± 79.01pg/ml(median:139, first quartile:111, third quartile:181), while it was 123.58 ± 46.61 pg/ml(median:117, first quartile:98, third quartile:132) in the control patients. This result was a statistically significant difference (*P* = 0.03), but there were no significant differences in the serum PD-L1 concentration (157.89 ± 55.34 pg/ml(median:152, first quartile:116, third quartile:185) in TED patients and 152.58 ± 22.70 pg/ml(median:154, first quartile:131, third quartile:187) in control patients, *P* = 0.92).

**Table 1:** Characteristics of TED and control patients.

	TED (n = 44)	Control (n = 19)	Remarks
Age (years)	56.06 ± 14.35	50.68 ± 18.29	*P* = 0.17
Gender			
Male	12	9	
Female	32	10	*P* = 0.15

TED, thyroid eye disease.

### 3.2 Immune checkpoint molecules before and after steroid pulse therapy

**Figure 3:**
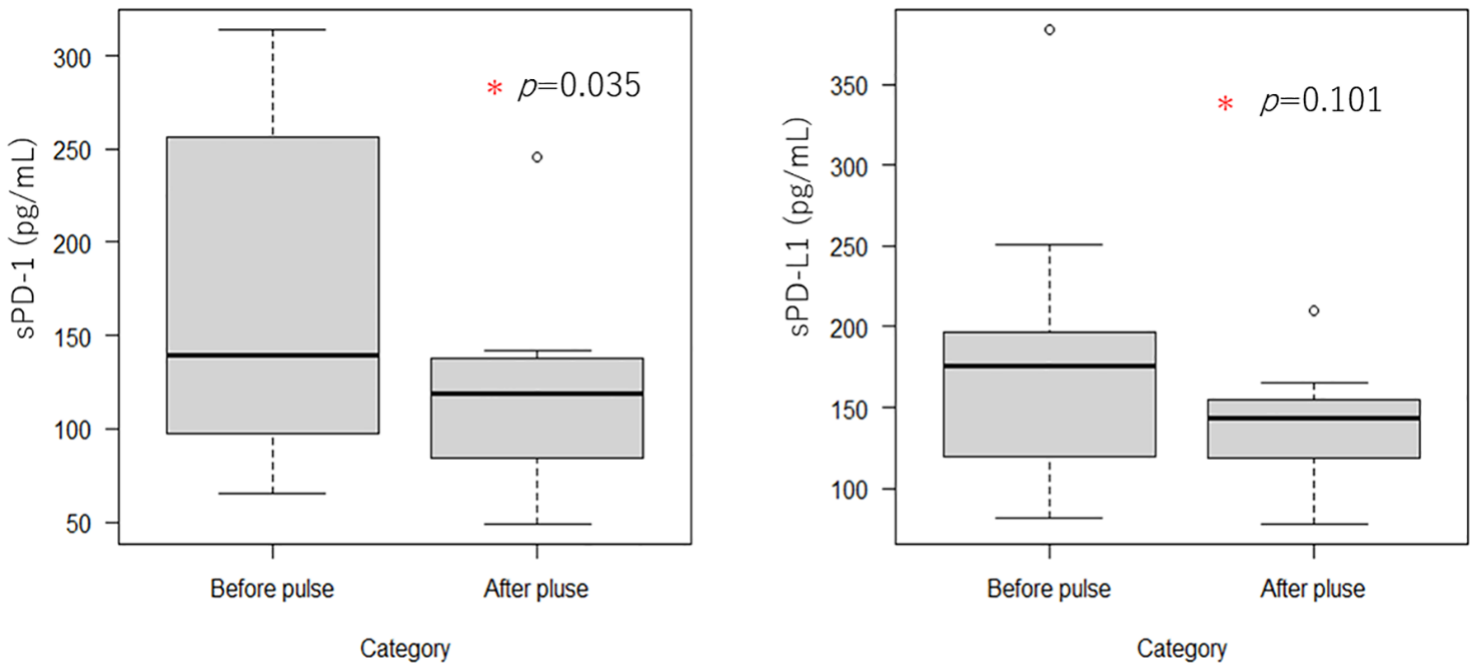
Boxplots of serum PD-1 and PD-L1 concentrations before and after steroid pulse therapy (n = 16). Serum PD-1 levels were significantly reduced by steroid pulse therapy in TED patients. In contrast, serum PD-L1 also tended to decrease, although there were no significant differences.

We collected blood from 16 TED patients who underwent steroid pulse therapy after half a year and then compared the serum immune checkpoint molecules before and after the steroid pulse therapy (**Figure 3**). The concentration of PD-1 before the steroid pulse treatment was 171.83 ± 85.34 pg/ml, while it was 116.19 ± 44.72 pg/ml in the after-steroid pulse treatment group, which was a statistically significant difference (*P* = 0.035). The concentration of PD-L1 was also higher before the steroid pulse treatment compared to after the treatment (173.50 ± 71.36 pg/ml versus 139.38 ± 28.89 pg/ml), but there was no statistically significant difference between the two groups (*P* = 0.101). **Figure 3** shows the scatterplots of the serum PD-1 and PD-L1 concentrations before and after the steroid pulse treatment.

### 3.3 Comparison of flow cytometry between the TED and control patients

Flow cytometric analysis was additionally performed in the 16 TED and 7 control patients. **Table 2** shows the results of the PD-1 mean fluorescence index (MFI) ratio and percentage of PD-1 expression on T cells for the flow cytometry analysis. **Figure 1** shows examples of the flow cytometry result for the TED and control patients. As an example, we show the results of flow cytometry analysis of a TED patient and a control patient. **Figures 1A–D** show the ratio of MFI of effector T cells to naive T cells in each T cell type (effector T cells and naive T cells were distinguished by the presence/absence of CD45RA expression). **Figure 1A** shows the MFI of CD4+ T cells, **Figure 1B** shows the MFI of CD4+ Foxp3high T cells, **Figure 1C** shows the MFI of CD4+ CD127-CD25+ T cells, and **Figure 1D** shows the MFI of CD8+ T cells. The upper rows [(a), (c), (e), (g)] of each figure show the MFI and MFI ratio of TED patients, and the lower rows [(b), (d), (f), (h)] show the MFI and MFI ratio of the control group.

**Table 2:** Results of PD-1 MFI ratio and percentage of PD-1 expression on T cells in flow cytometry analysis with TED (n = 16) and control (n = 7) patients.

	TED	Control	Remarks
MFI			
CD4+ T cells	1.23 ± 0.09	1.24 ± 0.16	*P* = 0.97
Foxp3high CD45RA- of CD4+ T cells	2.01 ± 0.51	1.49 ± 0.24	*P* = *0.004*
CD127- CD25+ of CD4+ T cells (Tregs)	1.65 ± 0.27	1.35 ± 0.10	*P = 0.001*
CD8+ T cells	1.17 ± 0.11	1.16 ± 0.17	*P* = 0.93
PD-1 expression (%)			
CD4+ T cells	12.89 ± 4.49	6.02 ± 2.17	*P* < 0.001
Foxp3high CD45- T cells	28.03 ± 13.95	12.47 ± 4.99	*P* = *0.001*
CD8+ T cells	17.89 ± 10.68	11.07 ± 5.82	*P* = 0.09

TED, thyroid eye disease.

MFI, mean fluorescence intensity. Italics indicate items for which significant differences were found.

About PD-1 MFI ratio, Foxp3high CD45RA-/Foxp3- CD45RA+ of CD4+ T cells and CD127- CD25+ (Treg)/CD4+ CD45RA+ of CD4+ T cells showed statistically significant differences between TED and control patients (2.01 ± 0.51 versus 1.49 ± 0.24, *P* = 0.004 and 1.65 ± 0.27 versus 1.35 ± 0.10, *P =* 0.001, respectively). These results mean that among CD4+ T cells, there is a significant difference between TED and control patients regarding MFI of Foxp3high CD45RA- T cells relative to Foxp3- CD45RA+ T cells. And among CD4+CD25+CD127- T cells, there is a significant difference about the MFI of Treg relative to CD45RA+ T cells between TED and control patients. However, there were no statistically significant differences about the MFI ratio on CD3+/CD45RA+ of CD4+ T cells and CD3+/CD45RA+ of CD8+ T cells (1.23 ± 0.09 versus 1.24 ± 0.16, *P* = 0.97 and 1.17 ± 0.11 versus 1.16 ± 0.17, *P* = 0.93, respectively).

We also compared the percentage of the PD-1 expression on the T cells during the flow cytometry analysis between the TED and control patients. There was a statistically significant difference (*P* < 0.001) for the CD4+ T cells. The PD-1 expression in TED was 12.89 ± 4.49, while it was 6.02 ± 2.17 in control patients. Regarding the Foxp3high CD45- T cells, the PD-1 expression in TED was 28.03 ± 13.95, while it was 12.47 ± 4.99 in the control patients. This difference was statistically significant (*P* = 0.001). However, for the CD8+ T cells, there was no significant difference in the PD-1 expression in TED 17.89 ± 10.68 as compared to 11.07 ± 5.82 in the control patients, (*P* = 0.09).

We also performed multiple regression analyses for the MFI and PD-1 expression of each T cell in conjunction with age, gender, and patient type (TED or control patient, **Table 3**). About the MFI of Foxp3high CD45RA- of the CD4+ T cells and CD127- CD25+ of the CD4+ T cells (Tregs), where a significant difference was found between TED and control patients earlier, there was a significant difference for the patient type (TED or control patient), although there were no significant differences observed for the age and gender (**Tables 3B, C**). For the PD-1 expression on the CD4+ T cells, in addition to the significant difference for the patient type, there was also a significant difference for gender (**Table 3E**). Furthermore, for the PD-1 expression on the Foxp3high CD45- T cells, while there was also a significant difference in patient type, no significant difference observed for the age and gender (**Table 3F**). Therefore, among the Foxp3-expressing T cells, there was a significant increase in the PD-1 expression in TED as compared to the control patients.

**Table 3:** Results of multivariate regression analysis: correlation with clinical factors between age, gender, and patient type (TED or control patient).

Variable	Regression coefficient	95%CI	*P*-value
A. MFI for the CD4+ T cells.			
Age	0.006	0.003 0.009	*<0.001*
Gender	-0.138	-0.214 -0.062	*0.001*
TED/Control	0.049	-0.047 0.145	0.30
B. MFI for the Foxp3high CD45RA- of the CD4+ T cells.			
Age	-0.008	-0.026 0.009	0.32
Gender	-0.166	-0.615 -0.283	0.45
TED/Control	-0.747	-1.314 - -0.180	*0.01*
C. MFI for the CD127-CD25+ of the CD4+ T cells (Tregs).			
Age	0.003	-0.006 - 0.011	0.49
Gender	-0.170	-0.394 - 0.055	0.13
TED/Control	-0.316	-0.599 - -0.033	*0.03*
D. MFI for the CD8+ T cells.			
Age	0.002	-0.003 - 0.007	0.36
Gender	-0.045	-0.178 - 0.087	0.48
TED/Control	0.014	-0.053 - 0.182	0.86
E. PD-1 expression on the CD4 T cells.			
Age	0.068	-0.062 - 0.197	0.29
Gender	-4.769	-8.144 - -1.394	*0.008*
TED/Control	-7.664	-11.922 - -3.406	*0.001*
F. PD-1 expression on the FOXP3 high CD45- T cells.			
Age	-0.074	-0.398 - 0.546	0.75
Gender	-5.261	-17.575 - 7.053	0.38
TED/Control	-16.458	-31.994 - -0.921	*0.04*
G. PD-1 expression on the CD8 T cells.			
Age	0.137	-0.225 - 0.498	0.44
Gender	-5.948	-15.374 - 3.478	0.20
TED/Control	-6.891	-18.784 - 5.002	0.24

Italics indicate items for which significant differences were found.

## 4 Discussion

Thyroid eye disease (TED) is an eye disease that often presents with thyroid dysfunction. While this is the most common hyperthyroid manifestation of Graves’ disease, it can also occur in euthyroid and, conversely, in hypothyroidism (1, 18). Compressive optic neuropathy and corneal ulcers can occur in severe TED patients, with these conditions sometimes potentially sight threatening. Even in non-severe cases, diplopia may occur and worsen the QOL.

In 1992, PD-1 was discovered by Ishida et al. at Kyoto University and reported as being an apoptosis-associated gene (9). Subsequent studies by the same group showed that PD-1 expression is induced via signaling by antigen receptors on T and B lymphocytes (19). Moreover, they also subsequently reported that PD-1-deficient mice develop an autoimmune phenotype, with the results demonstrating that the PD-1 identified was involved in the inhibition of immune responses (10). It is now known that the immune checkpoint molecules are the inhibitory receptors that are expressed on the surface of the immune cells and regulate the immune responses to prevent these from becoming excessive (20). PD-L1 (a ligand for PD-1) is also an immune checkpoint molecule, and these molecules are thought to play an important role in the onset of autoimmune diseases (21).

Several reports have shown that serum immune checkpoint molecule concentrations are elevated in autoimmune diseases. Kobayashi et al. reported that in Sjögren’s syndrome, MFI of the PD-1 expression in salivary lymphocytes was significantly higher as compared to that in healthy controls and patients with rheumatoid arthritis or systemic lupus erythematosus (22). Oaks et al. additionally reported that immune checkpoints in autoimmune thyroid disease molecule (sCTLA-4) levels were higher as compared to that in the control group (23). However, definitive findings regarding the concentration of the immune checkpoint molecules in autoimmune thyroid diseases have yet to be obtained.

There are reports of cases of thyroid ophthalmopathy caused by immune checkpoint inhibitors. Min et al. reported a case of thyroid ophthalmopathy disease that developed in a patient with no history of thyroid disease after undergoing treatment with ipilimumab (Yervoy^®^: anti-CTLA-4 antibody) for malignant melanoma (12). Campredon et al. reported a case of thyroid eye disease-like symptoms in a person with no history of thyroid disease that was caused by a nivolumab (Opdivo^®^: anti-PD-1 monoclonal antibody) treatment for non-small cell lung cancer (13). Although thyroid stimulating hormone receptor antibody (TRAb) and TSAb were not measured in the above two cases, there have been some reports on worsening thyroid eye disease that occurred due to PD-1/PD-L1 inhibitor treatments. Thus, this suggests that immune checkpoint molecules may be associated with inflammation in thyroid ophthalmopathy.

In the present study, serum PD-1 concentrations in TED patients were significantly increased as compared to control patients (*P* = 0.03). When TED patients underwent steroid pulse therapy, serum PD-1 significantly decreased before and after treatment (*P* = 0.01). Furthermore, PD-L1 also tended to decrease after steroid pulse therapy, although there was no significant difference (*P* = 0.055). The flow cytometry results showed that the MFI of PD-1 on Foxp3high CD4+ CD45RA- T cells and CD127- CD25+ CD4+ T cells (Treg) was significantly higher in the TED group, and the PD-1 expression rate in Foxp3high T cells was also significantly higher. In other words, it can be considered that expression of PD-1 on Foxp3 positive T cells is increased not only on surface but also in these cells. When taking into consideration both the results of the present study and several past reports, these findings suggest that PD-1 is involved in TED inflammation and that patients with untreated TED may have elevated serum PD-1/PD-L1 levels.

It remains unclear why serum PD-1 levels are elevated in untreated TED patients, but there are several hypotheses that might explain these findings. First, these elevations may be due to the action of insulin-like growth factor 1 (IGF-1), which has also been involved in the pathogenesis of TED.

As mentioned above, the relevance of IGF-1 receptor (and thyroid-stimulating hormone receptor: TSHR) in TED is known, and Salminen et al. reported that the insulin/IGF-1-induced STAT3 pathway may cooperate with SMAD3 and NF-κB signaling to promote the activation of immunosuppressive cells, so that the expression levels of PD-1 and PD-L1 might increase (24).

The secondary hypothesis involves the exhaustion that occurs in T cells. When T cells are exposed to persistent inflammatory signals, such as chronic infection or cancer, these T cells can convert to an exhausted status, which is characterized by a loss of effector functions and memory T cell properties, and by the expression of multiple inhibitory receptors (25–27). When T cells become exhausted, it then becomes difficult to control inflammation, infection, and tumors. Several previous studies have implicated the PD-1/PD-L1 pathway in T cell exhaustion, which occurs when T cells are chronically exposed to high antigen loads (28). It has also been suggested that blocking the PD-1/PD-L1 interaction restores the exhausted CD8 cells. Thus, PD-1 is thought to be related to T cell exhaustion. This change has been previously reported in CD8+ T cells, and antigen specific CD4+ T cells, which also can become exhausted T cells during chronic infection. However, it should be noted that exhaustion of CD4 T cells differs from that of CD8 T cells, as there are some differences in the patterns of molecules, such as inhibitory receptors and transcription factors (25).

**Figure 4:**
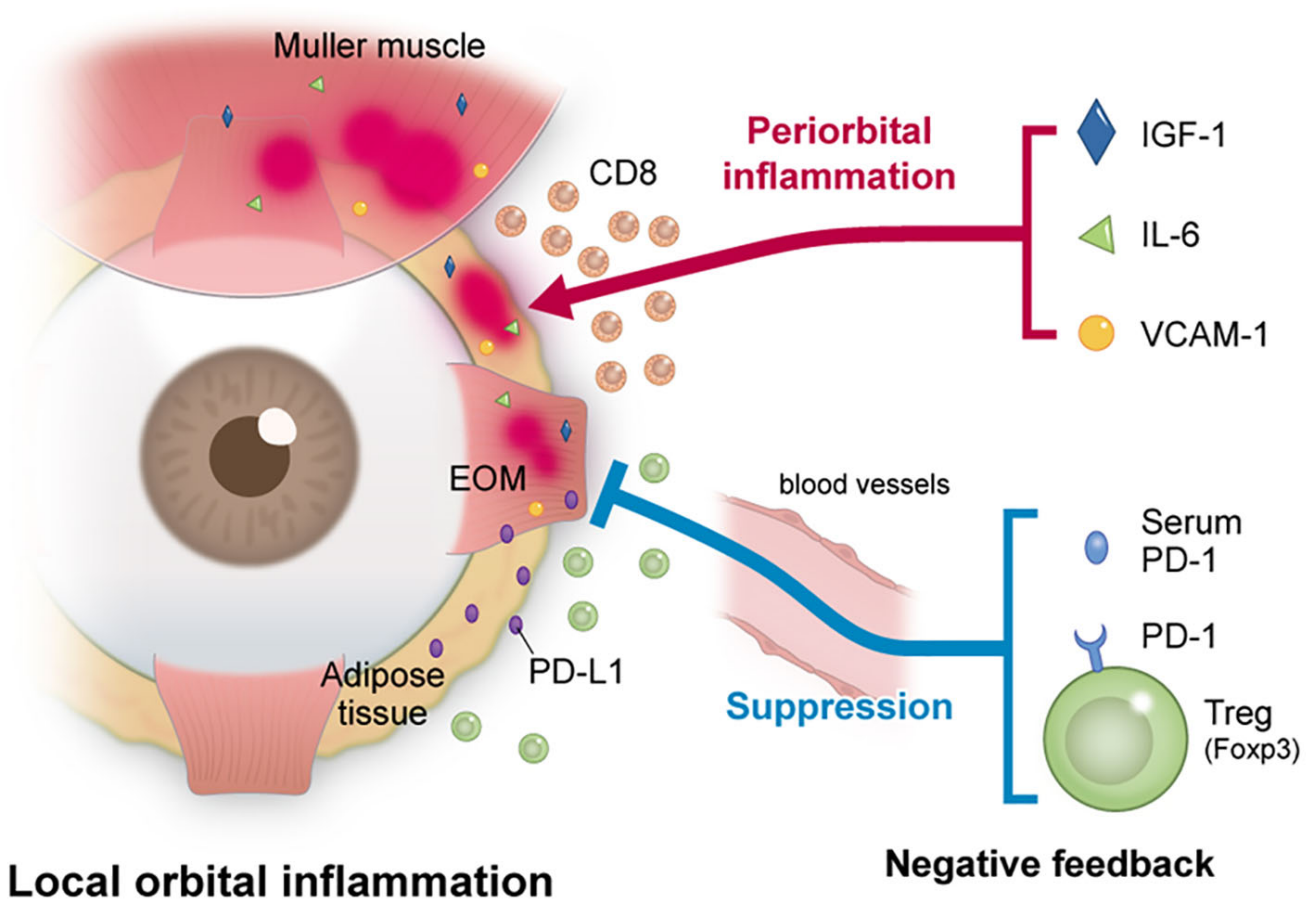
The relationship between inflammation and inflammation suppression in TED patients. In TED, orbital inflammation was induced by IGF-1 and various cytokines. The results of the present study suggest that the expression of PD-1 in the serum and on Tregs increased and led to a suppression of orbital inflammation.

Thirdly, it is conceivable that the expression level of PD-1 was not increased due to exhaustion, but rather due to suppression of the inflammation. Several previous reports have discussed the orbital infiltration of T cells in TED as below. Wiersinga et al. reported that examination of the T cells infiltrating the orbit were mostly CD4+ and CD8+ T cells, with only a few B cells in TED patients (29). Förster et al. collected orbital adipose or connective tissue from 6 TED patients who underwent orbital decompression surgery, and they made 18 T cell lines (TCL). They reported that the TCL of all but one were composed primarily of CD4+ T cells, with 10 of the 18 strains primarily composed of CD4+ T cells, while the other strains were primarily composed of CD8+ T cells (30). PD-1 is expressed on CD4+ T cells, CD8+ T cells, NKT cells, B cells and monocytes, while both PD-L1 and PD-1 are expressed on CD4+ CD25+ T cells, such as regulatory T cells (31). The results of this study showed that PD-1 increased in untreated TED as compared to the control group, while PD-1 decreased in TED after steroid pulse treatment. Flow cytometry examination additionally showed that the MFI of Foxp3high CD45RA- of the CD4+ T cells and CD127- CD25+ of the CD4+ T cells, and the PD-1 expression rate of the CD4+ T cells and Foxp3high CD45- T cells were significantly higher as compared to those of the control patients. In other words, PD-1 expression was high in untreated TED patients, and when taking into consideration that the PD-1 expression decreased due to the improvement of orbital inflammation, this suggests that Treg infiltrated into the orbital region to suppress the inflammation in TED patients. The findings of this study indicated that PD-1 expression was increased in Foxp3high T cells such as Tregs, which may have led to the suppression of orbital inflammation. **Figure 4** shows orbital inflammation in TED patients and the anti-inflammatory effect of Tregs.

There were some limitations in the present study. First, this study was a retrospective study. Second, the number of cases may not have been sufficient for both the TED and control patient groups. However, even though the number of cases was small, we did find that the PD-1 expression level was increased in thyroid eye disease. This will need to be further verified in an additional study with an increased number of cases. Third, the target patient population in this study was limited to Asians. Thus, similar verifications will need to be conducted for other races as well.

The present study found that the PD-1 expression levels on T cells increased in TED patients. In other words, immune checkpoint molecules are thought to be involved in TED inflammation, and thus, this can potentially be used to predict prognosis and lead to the suppression of orbital inflammation via immune checkpoint molecules.

In conclusion, serum PD-1 levels were significantly higher in untreated TED patients as compared to non-TED patients, with steroid pulse therapy significantly reducing the PD-1 levels in TED patients. Flow cytometry examinations demonstrated that the PD-1 expression rate of CD4+ T cells and Foxp3high CD45- T cells were significantly higher in TED patients as compared to control patients. Further investigations of the relationship between ocular inflammation and immune checkpoint molecules in TED could potentially lead to elucidation of the pathology and development of new treatments in the future.

## Data Availability

The original contributions presented in the study are included in the article/supplementary material. Further inquiries can be directed to the corresponding author.
